# Genetic engineering of human NK cells to express CXCR2 improves migration to renal cell carcinoma

**DOI:** 10.1186/s40425-017-0275-9

**Published:** 2017-09-19

**Authors:** Veronika Kremer, Maarten Ligtenberg, Rosa Zendehdel, Christina Seitz, Annet Duivenvoorden, Erik Wennerberg, Eugenia Colón, Ann-Helén Scherman-Plogell, Andreas Lundqvist

**Affiliations:** 1 Department of Oncology-Pathology, Karolinska Institutet, Stockholm, Sweden; 2 Department of Molecular Oncology, The Netherlands Cancer Institute, Amsterdam, Netherlands; 3 Department of Medicine Solna, Karolinska Institutet, Stockholm, Sweden; 4 Department of Radiation Oncology, Weill Cornell Medicine, New York, NY USA; 5 Department of Oncology-Pathology, Stockholm South General Hospital, Stockholm, Sweden; 6 Department of Woman and Child Health, Karolinska Institutet, Stockholm, Sweden; 7 Department of Urology, Stockholm South General Hospital, Stockholm, Sweden; 8 Cell Therapy Institute, Nova Southeastern University, Fort Lauderdale, FL USA

**Keywords:** NK cells, Chemokines, CXCR2, Renal cell carcinoma, Adoptive cell therapy

## Abstract

**Background:**

Adoptive natural killer (NK) cell transfer is being increasingly used as cancer treatment. However, clinical responses have so far been limited to patients with hematological malignancies. A potential limiting factor in patients with solid tumors is defective homing of the infused NK cells to the tumor site. Chemokines regulate the migration of leukocytes expressing corresponding chemokine receptors. Various solid tumors, including renal cell carcinoma (RCC), readily secrete ligands for the chemokine receptor CXCR2. We hypothesize that infusion of NK cells expressing high levels of the CXCR2 chemokine receptor will result in increased influx of the transferred NK cells into tumors, and improved clinical outcome in patients with cancer.

**Methods:**

Blood and tumor biopsies from 14 primary RCC patients were assessed by flow cytometry and chemokine analysis. Primary NK cells were transduced with human CXCR2 using a retroviral system. CXCR2 receptor functionality was determined by Calcium flux and NK cell migration was evaluated in transwell assays.

**Results:**

We detected higher concentrations of CXCR2 ligands in tumors compared with plasma of RCC patients. In addition, CXCL5 levels correlated with the intratumoral infiltration of CXCR2-positive NK cells. However, tumor-infiltrating NK cells from RCC patients expressed lower CXCR2 compared with peripheral blood NK cells. Moreover, healthy donor NK cells rapidly lost their CXCR2 expression upon in vitro culture and expansion. Genetic modification of human primary NK cells to re-express CXCR2 improved their ability to specifically migrate along a chemokine gradient of recombinant CXCR2 ligands or RCC tumor supernatants compared with controls. The enhanced trafficking resulted in increased killing of target cells. In addition, while their functionality remained unchanged compared with control NK cells, CXCR2-transduced NK cells obtained increased adhesion properties and formed more conjugates with target cells.

**Conclusions:**

To increase the success of NK cell-based therapies of solid tumors, it is of great importance to promote their homing to the tumor site. In this study, we show that stable engineering of human primary NK cells to express a chemokine receptor thereby enhancing their migration is a promising strategy to improve anti-tumor responses following adoptive transfer of NK cells.

**Electronic supplementary material:**

The online version of this article (doi:10.1186/s40425-017-0275-9) contains supplementary material, which is available to authorized users.

## Background

Natural killer (NK) cells have received renewed attention as an immunotherapeutic treatment against cancer in the recent years. The advantage of NK cells is that they can kill a wide spectrum of tumor cells without the requirement for specific antigen recognition. In different animal models, NK cells have been shown to eradicate engrafted tumors and, in particular, metastases. Adoptive transfer of NK cells has successfully been used to treat patients with leukemia and other hematological cancers [1–3]. However, clinical responses in patients with solid tumors treated with adoptively infused NK cells have so far not been beneficial [4, 5]. A potential limiting factor for the success of adoptive NK cell therapy is the inefficient migration of NK cells to the tumor site [6–9], which may partially explain the poor clinical responses in cancer patients. Several studies have correlated high numbers of tumor-infiltrating NK cells with a good prognosis in various cancers [10–12]. Renal cell carcinoma (RCC) is particularly noteworthy as multiple independent studies have associated intratumoral NK cell infiltration with increased survival of metastatic RCC patients [13–15], whereas CD8+ T cell infiltration has been indicative of a poor prognosis [16]. Therefore, improving NK cell infiltration into RCC tumors is an attractive therapeutic option to potentiate the success of NK cell-based therapies and eventually improve clinical responses.

Chemokines are small secreted proteins that control the recruitment of immune cells with corresponding chemokine receptors to sites of infection, tumors or other tissues. The presence of specific chemokines in the tumor microenvironment, including ligands for the chemokine receptors CXCR3 and CX3CR1, has been shown to promote intratumoral infiltration of T and NK cells and correlate with increased survival in a variety of cancers [17–19]. We have recently shown a strategy to enhance the migration of CXCR3-positive human NK cells to melanoma tumors by stimulating local CXCL10 secretion as CXCR3 ligands are not usually present at the tumor site [20]. Ligands for the chemokine receptor CXCR2, on the other hand, are readily secreted by a variety of solid tumors, including RCC [21], to promote angiogenesis, tumor growth and metastasis.

The CXCR2 chemokine receptor is expressed on a number of leukocytes, most prominently on neutrophils and monocytes, facilitating their infiltration into solid tumors [22]. While CXCR2 is present on peripheral blood NK cells, we found that its expression is rapidly lost during their in vitro culture. We show here that genetic modification of human primary NK cells to re-express CXCR2 improves their ability to specifically migrate along a tumor-derived chemokine gradient resulting in increased killing of target cells. In addition, while their functionality remains unchanged, NK cells incorporating the CXCR2 gene obtain increased adhesion properties. Thus, re-expression of CXCR2 through genetic engineering of ex vivo expanded NK cells represents a novel strategy to improve anti-tumor responses following adoptive transfer of NK cells.

## Methods

### Patient samples

Blood and tumor biopsies were collected from 12 clear cell RCC patients, one papillary type 2 and one chromophobe RCC patient undergoing nephrectomy at the time of surgery and 1 to 2 months after during the period of April 2014 till October 2015. The diagnosis was confirmed histologically, and the cellular grading was determined according to Fuhrman. All clear cell and papillary RCC patients were Fuhrman grade II-IV. Plasma was obtained by centrifugation of the blood samples and stored at −20 °C until analysis. Whole blood was stained with appropriate antibody mixes and analyzed by flow cytometry. Tumor samples were snap-frozen in liquid nitrogen and stored at −80 °C until tissue lysates were prepared using CelLytic MT extraction buffer (Sigma) following the manufacturer’s protocol.

### Cell lines

The primary RCC cell lines TINCA1, TINCA3, TINCA7, and MAR were established from surgically resected tumor specimens. The MAR cell line was kindly provided by Dr. Richard Childs (National Institutes of Health). The RCC cell lines ACHN, Caki-2, A498 and the myelogenous leukemia cell line K562 were obtained from the American Type Culture Collection (ATCC). Caki-1 and 786-O cells were kindly provided by Prof. Barbara Seliger (Martin Luther University Halle-Wittenberg, Germany). All cell lines were maintained in RPMI1640 or DMEM (Thermo Fisher Scientific) supplemented with 10% FBS (Thermo Fisher Scientific). The TINCA cell lines were cultured with 20% FBS. Cell lines (ACHN, A498, Caki-2, and 786-O) were authenticated using the AmpFLSTR Identifiler PCR Amplification Kit (Thermo Fisher Scientific).

### Expansion and retroviral transduction of NK cells

NK cells were isolated from PBMCs and expanded using a GMP-compliant protocol with irradiated EBV-LCL feeder cells as previously described [23]. Retroviral particles containing the pMSGV1-CXCR2 vector were recovered from supernatant of confluent cultures of PG13 packaging cells, kindly provided by Dr. Patrick Hwu (University of Texas M.D. Anderson Cancer Center, USA). Retrovirus containing the vector pMSGV1-NGFR-N which encodes the human nerve growth factor receptor gene was used as control. NK cells expanded for eight to 10 days and confirmed to be pure from feeder cells by flow cytometry were transduced using RetroNectin reagent (Takara Bio) following the manufacturer’s protocol. Briefly, viral supernatant was bound to RetroNectin-coated 6-well plates by 2 hour centrifugation at 32 °C at 2000 x g. After virus removal, NK cells were added to the wells at 0.5 × 10^6^/mL in X-Vivo 20 medium containing 10% human AB serum and 1000 IU/mL IL-2 and centrifuged at 1000 x g for 10 min. Viral spinoculation was repeated on the following day to improve transduction efficiency. The next day, NK cells were pooled and cultured at a concentration of 1 × 10^6^/mL supplemented with 500 IU/mL IL-2 for two to 3 days. For the migration experiments, transgene-positive NK cells were isolated by positive selection with anti-APC beads (Miltenyi Biotech) with >90% purities on average.

### Chemokine analysis

Primary RCC cell line supernatants were collected from 24-h cultures of 2 × 10^5^ cells/mL in 24-well plates. Supernatants from established RCC cell lines and MAR were generated by culturing 4 × 10^5^ cells/mL in serum-free RPMI1640 for 6 hours after they had attached overnight in their respective medium in 24-well plates. The levels of chemokines in RCC patient plasma, lysates from tumor specimens and in the RCC cell line supernatants were quantified using the Bio-Plex Pro Human Chemokine 40-plex Panel (Bio-Rad) according to the manufacturer’s instructions. The analysis was performed using a Milliplex Magpix System with xPONENT 4.2 control software (Merck Millipore) and Bio-Plex Manager 6.1 analysis software (Bio-Rad). The concentrations of CXCL1 and CXCL8 in RCC tumor supernatants were additionally analyzed by ELISA (Bio-Techne) according to the manufacturer’s protocols.

### Chromium release assay

To determine the cytotoxic activity of NK cells, K562, ACHN and Caki-2 cells were labeled with ^51^Cr and co-cultured with NK cells at different effector-to-target ratios for 5 or 20 h as previously described [20]. Specific lysis was calculated as the percentage of ^51^Cr release using the following formula: % specific lysis = (sample release – spontaneous release)/(maximum release – spontaneous release) × 100%.

### Flow cytometry

Stainings were performed using appropriate combinations of the following anti-human monoclonal antibodies: CD56-FITC, CD16-FITC, CD107a-FITC, CD3-PE, CXCR2 (CD182)-APC, NKp46-APC, CD11b-PE-Cy7 (BD Biosciences), DNAM-1-FITC, TRAIL-PE, FasL (CD178)-PE, CD11a-PE, CXCR2 (CD182)-PE, CD56-PE-Cy7, CXCR2 (CD182)-APC, NGFR-N (CD271)-APC, NKp30-APC, IFN-γ-APC, CD56-Pacific Blue, CD19-Brilliant Violet 570, CD3-Brilliant Violet 605 (Biolegend), CD2-APC (ImmunoTools), CD3-Pacific Orange (Thermo Fisher Scientific), and active conformation LFA-1 (CD11/CD18, mAb24) (Hycult Biotech) labeled with Zenon Pacific Orange Mouse IgG1 Labeling kit (Thermo Fisher Scientific). 7-AAD (BD Biosciences), LIVE/DEAD Fixable Near-IR and LIVE/DEAD Fixable Aqua Dead Cell Stain kits (Thermo Fisher Scientific) were used to exclude dead cells. Data were acquired on a LSR II (BD Biosciences) or a NovoCyte flow cytometer (ACEA Biociences) and analyzed using FlowJo software (TreeStar).

NK cells (6 × 10^5^ cells/mL) were co-cultured with K562 cells at a ratio of 1:1 and with RCC cells at a ratio of 2:1 to 1:1 for 6 h at 37 °C and stained for CD107a and IFN-γ to evaluate degranulation and IFN-γ production by flow cytometry.

For NK cell proliferation assays, NK cells were incubated with 5 μM carboxyfluorescein succinimidyl ester (CellTrace CFSE cell proliferation kit, Thermo Fisher Scientific) in PBS for 15 min at 37 °C, pelleted and incubated for additional 30 min in medium. Labeled NK cells were plated in 96-well plates, stimulated with 500 U/mL IL-2 and incubated at 37 °C. IL-2 was replenished every 3 days. After 7 days, NK cells were stained with surface antibodies and proliferation was evaluated by flow cytometry.

### Conjugate formation assay

NK cells and K562 cells were labeled with 0.36 μM CFSE or with 5 μM CellTracker Violet BMQC dye (Thermo Fisher Scientific) following the manufacturer’s instructions. In addition, NK cells were stained with APC-labeled anti-CXCR2 or anti-NGFR antibodies and, in some experiments, subsequently pre-incubated with 10 mg/ml anti-CD11a for 20 min at 4 °C. Next, NK cells (1 × 10^5^) and K562 cells (1 × 10^5^) were mixed at an effector-to-target ratio of 1:1 in a final volume of 200 μL of X-Vivo 20 medium with 10% human AB serum, centrifuged at 4 °C for 1 min at 20 x g, and incubated in a 37 °C water bath for 10 min. Reactions were stopped by adding 0.5% paraformaldehyde. Conjugate formation was analyzed by flow cytometry, and the percentage of NK cells in conjugates was calculated as the ratio of double positive events to total effector cell events.

### Calcium mobilization assay

NK cells were loaded with 20 μM Fluo-3, AM (Thermo Fisher Scientific) in the presence of 0.1% (*w*/*v*) Pluronic F-127 (Thermo Fisher Scientific) in HBSS with Ca^2+^/Mg^2+^ (Thermo Fisher Scientific) for 1 h at room temperature (RT). Cells were washed once and stained with APC-labeled anti-CXCR2 or anti-NGFR antibodies. After washing, 10 times diluted aliquots of cell suspensions were kept at RT in the dark until analysis. Transient increase in Fluo-3 fluorescence upon intracellular calcium release in the presence of indicated stimuli was measured at 530/30 nm using a LSR II flow cytometer for 300 s. Ionomycin (200 ng/mL) was used as a positive control for dye loading. Fluo-3 relative fluorescence units (RFU) were calculated as mean fluorescence intensity (MFI) of Fluo-3 normalized to the MFI mean of the baseline obtained in the initial 30 s of the recording prior to the addition of stimuli. The magnitude of the response after addition of the stimuli was calculated as the normalized area under curve (AUC).

### Chemotaxis assays

To measure NK cell migration in real time, NK cells (5 × 10^3^) were placed in 60 μL medium containing 0.5% FBS into the upper chamber of an IncuCyte ClearView chemotaxis plate (Essen Biosciences), which had been coated with fibronectin (5 μg/mL in 0.1% BSA) for 30 min at 37 °C and 30 min at RT. NK cells were then allowed to settle for 1 h at RT. Next, the insert plate was transferred onto the reservoir plate pre-filled with 200 μL/well serum-free medium with or without recombinant CXCL1, CXCL2, CXCL3 (PeproTech) at 50 ng/mL and CXCL8 (PeproTech) at 100 ng/mL. The assembled plate was then placed into the IncuCyte® ZOOM instrument inside a 37 °C incubator. The camera was set to take images of the top side of each well every 2 h. Migration was quantified by the IncuCyte® analysis software using the chemotaxis top mask as total area occupied by NK cells on the top well surface normalized to the occupied area at time point *t* = 0 h (*n* = 4 per condition).

For transwell assays, 600 μL serum-free medium containing recombinant CXCL1, CXCL2, CXCL3, CXCL5, CXCL6, CXCL7 and CXCL8 (Peprotech) at 50 ng/mL or conditioned medium from the indicated RCC cell lines (obtained as described above) was placed in the lower chamber of a 24-well transwell plate (Corning). NK cells (2.5 × 10^5^) were added in 100 μL serum free-medium to the upper chamber (5-μm pore size), and the plates were incubated for 2 h at 37 °C. To block NK cell migration, NK cells were pre-incubated with 1 μM of the selective non-peptide CXCR2 inhibitor SB225002 (Cayman Chemical) for 30 min at 37 °C. To distinguish between chemotaxis and chemokinesis, CXCR2 ligands were added to the upper and lower chambers in equal concentrations. The number of NK cells that migrated to the lower chamber was determined by automated counting of cells in a 200 μL aliquot using a NovoCyte flow cytometer. Data are presented as percentage of migration based on total cell input.

To evaluate NK cell-mediated cytotoxicity after migration, ^51^Cr-labeled K562 cells (7 × 10^5^) were placed in the lower transwell chamber in serum-free medium with or without CXCR2 ligands. After the NK cells were allowed to migrate for 2 h at 37 °C, the inserts were removed and FBS was added to 10%. Supernatant was harvested from each well in quadruplicates after an additional 4-h incubation (6 h in total).

### Statistics

Paired Student’s *t* tests were performed for individual comparisons of two paired groups after confirming normal distribution of the data. Correlation analysis was performed using Pearson correlation for normally distributed data. For multiple matched group comparisons, one-way or two-way repeated measures ANOVA was applied. For all statistical analyses the Prism software version 6 and 7 (GraphPad Software) was used. Significance was defined by *p*-values less than 0.05 using a two-tailed test. *, *P* < 0.05; **, *P* < 0.01; ****, *P* < 0.0001.

## Results

### RCC tumors express CXCR2 ligands, while tumor-infiltrating NK cells reduce CXCR2 expression

**Fig. 1:**
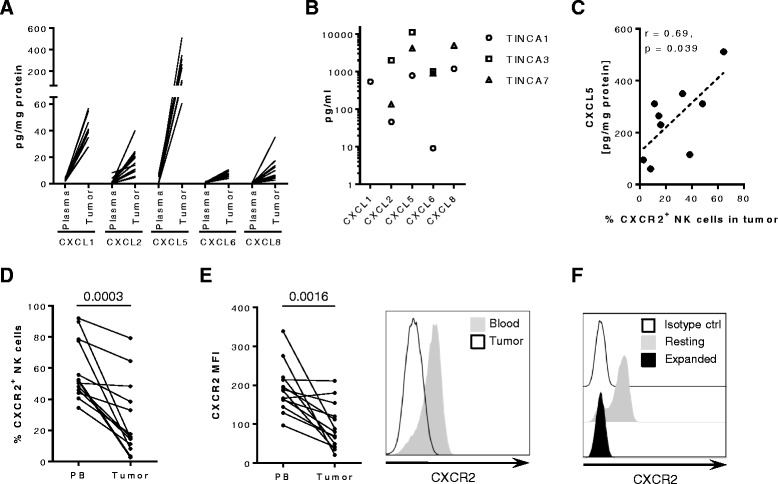
Expression of CXCR2 on NK cells and its ligands on RCC tumors. **a** Expression of CXCR2 ligands in the plasma and tumor lysate of patients with primary RCC relative to mg total protein (*n* = 14). Samples were analyzed using Bio-Plex Pro Human Chemokine 40-plex panel. **b** Expression of CXCR2 ligands by primary low-passage (P1 or P3) RCC cell lines. CXCL1 production by TINCA3 and TINCA7 as well as CXCL8 production by TINCA3 were above the quantification limits of 13,990 pg/mL and 31,093 pg/mL, respectively. **c** Pearson correlation of CXCL5 levels in tumor lysate in patients with primary RCC and frequency of intratumoral CXCR2-positive NK cells (*n* = 9). **d** Frequency and (**e**) levels of CXCR2 expression by NK cells in peripheral blood (PB) and primary tumors of RCC patients (*n* = 13). A representative histogram from patient RCC007 is shown. **f** Flow cytometry analysis of CXCR2 expression by healthy donor peripheral blood non-activated NK cells and eight-day expanded NK cells. Results are representative of four experiments

Primary tumor tissues and plasma from 14 RCC patients that underwent nephrectomy were evaluated for the presence of cognate ligands for the chemokine receptor CXCR2 by Bio-Plex chemokine array (Fig. 1a). For CXCL1, CXCL2, CXCL6 and CXCL8, there was on average a 10- to 24-fold concentration gradient (per mg protein) between patient plasma and tumors. The greatest difference in average concentration between tumor and plasma was found for CXCL5 (186-fold gradient) as the chemokine was largely not detectable in patient plasma, while in tumor lysates, its concentration was highest of all analyzed CXCR2 ligands. However, CXCL5 was only detected in nine of the 14 tumor samples. The concentration of chemokines in the plasma one to two months after surgery did not significantly change compared with the concentration at surgery (data not shown). CXCR2 ligands were also secreted by the low passage (≤3 passages) RCC cell lines TINCA1, 3, and 7 established from three of the patient tumor samples (Fig. 1b). Furthermore, the presence of CXCR2-positive NK cells in the tumors significantly increased with higher concentrations of CXCL5 (*p* = 0.039), while total NK cell frequencies were comparable in CXCL5 high and low tumors (Fig. 1c and data not shown). This correlation was not observed for any of the other analyzed CXCR2 chemokines (data not shown). Overall, however, frequencies of CXCR2-positive NK cells were significantly lower in the tumors compared with peripheral blood (*p* = 0.0003) as were CXCR2 expression levels on those NK cells (*p* = 0.0016) (Figs. 1d-e). Moreover, we found that while human circulating NK cells from healthy donors expressed CXCR2 at a resting state, they rapidly down-regulated CXCR2 expression upon ex vivo activation and expansion (Fig. 1f and Additional file 1: Figure S1). Hence, adoptively transferred ex vivo activated or expanded NK cells are unlikely to migrate to the CXCR2-ligand gradient present at the tumor site.

### CXCR2 retroviral transduction does not alter the function of human primary NK cells

**Fig. 2:**
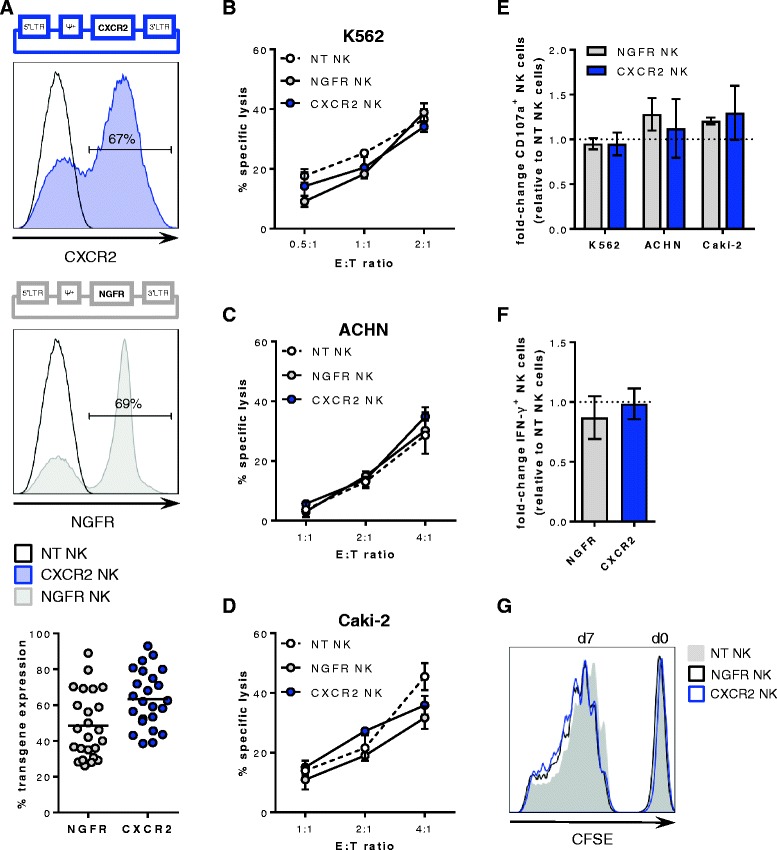
Retroviral transduction and functionality of NK cells. **a** Top and middle: Schematic representation of retroviral vectors containing human CXCR2 and NGFR, respectively, and representative histograms of the transgene expression on non-transduced (NT), CXCR2- and NGFR-transduced primary NK cells. Bottom: Flow cytometry analysis of CXCR2 and NGFR expression on NK cells after the transduction (*n* = 24). Horizontal bars represent the mean transduction efficiency. **b** NK cell-mediated cytotoxicity against K562 cells after 5 h. One of four representative experiments is shown. NK cell-mediated cytotoxicity against (**c**) ACHN and (**d**) Caki-2 cells after 20 h. One of five and three representative experiments is shown, respectively. **e** Flow cytometry analysis of NK cell degranulation after stimulation with K562 at a E:T ratio of 1:1 (*n* = 4) and with ACHN and Caki-2 at a E:T ratio of 2:1 to 1:1 (*n* = 3 for each cell line). **f** Flow cytometry analysis of NK cell IFN-γ production after stimulation with K562. Data in **e** and **f** are depicted as mean ± SEM of fold-change compared with non-transduced NK cells and analyzed with repeated measures one-way ANOVA. **g** Proliferation of non-transduced (NT), NGFR-transduced, and CXCR2-transduced NK cells after 7 days as assessed by CFSE staining. Results are representative of three experiments

In order to promote the migration of adoptively transferred ex vivo expanded NK cells to tumors that secrete CXCR2 chemokines, human primary NK cells were transduced with human CXCR2 using a Murine Stem Cell Virus-derived retroviral expression system. NK cell transductions with the nerve growth factor receptor (NGFR) were carried out to control for effects resulting from the insertion of the virus into the genome. Transgene expression ranged from 26 to 93% (Fig. 2a) and was stable over the course of the cell culture period of two to 3 weeks as well as after exposure to recombinant and RCC tumor-derived CXCR2 ligands (data not shown). Importantly, the transduction did not compromise the effector functions of the transduced NK cells. Upon co-culture with K562 cells, NK cell cytotoxicity (ranging from 11 to 45%), degranulation (ranging from 20 to 55%) as well as IFN-γ production (ranging from 5 to 11%) were similar in non-transduced, CXCR2- and NGFR-transduced NK cells (Fig. 2b, e-f and Additional file 2: Figure S2). Likewise, NK cell cytotoxicity against the RCC cell lines ACHN and Caki-2 (ranging from 13 to 79% and 19 to 45%, respectively) as well as degranulation (ranging from 2 to 15%) were not different in transduced compared with non-transduced NK cells (Fig. 2c-e). However, the RCC cells did not induce any detectable IFN-γ production (data not shown). Furthermore, the proliferation of transduced NK cells did not differ from non-transduced NK cells (Fig. 2g).

### NK cells incorporating the CXCR2 transgene have increased adhesion properties

**Fig. 3:**
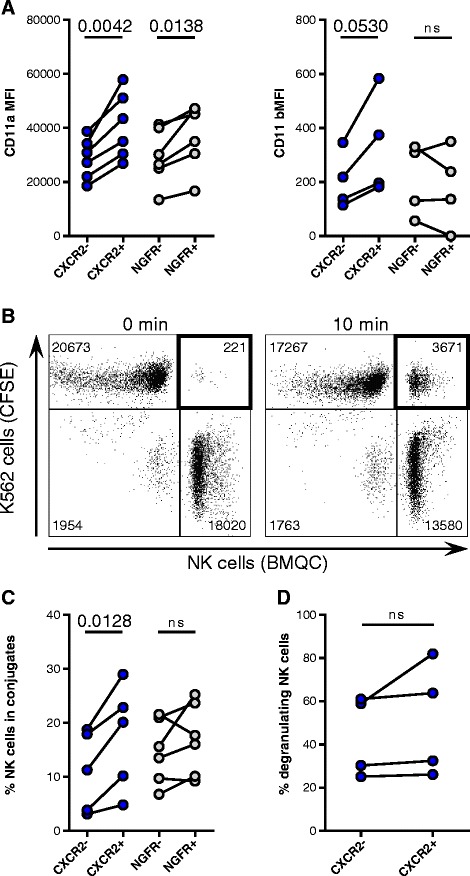
Adhesion of NK cells incorporating the transgenes. **a** Flow cytometry analysis of CD11a (*n* = 6) and CD11b (*n* = 4) expression on NK cells incorporating (CXCR2+ and NGFR+) and not incorporating the respective transgene (CXCR2- and NGFR-). **b** Representative dot plots depicting counts of collected events within the double-positive gate after 0-min and 10-min co-cultures of CFSE-labeled K562 cells and BMQC-labeled NK cells. **c** NK cells incorporating and not incorporating the CXCR2 (*n* = 5) or NGFR (*n* = 6) transgenes in conjugates with K562 cells after 10 min of co-culture. **d** Degranulation against K562 of CXCR2- and CXCR2+ NK cells, as assessed by flow cytometry (*n* = 4)

Although the expression of activating receptors was similar between transduced and non-transduced NK cells (data not shown), the expression of CD2 by CXCR2-transduced NK cells significantly increased compared with non-transduced cells (1.7-fold change, *p* = 0.0059) (Additional file 3: Figure S3). Overall, the expression of the β2 integrins LFA-1 (CD11a/CD18 heterodimer) and Mac-1 (CD11b/CD18 heterodimer) did not change on transduced cells (data not shown). However, CD11a levels on NK cells incorporating the CXCR2 and NGFR transgene (CXCR2+ and NGFR+) were 1.4-fold (*p* = 0.0042) and 1.3-fold (*p* = 0.0138) higher, respectively, compared with NK cells that had not incorporated the transgene (CXCR2- and NGFR-) (Fig. 3a). Similarly, CD11b expression increased 1.6-fold on CXCR2+ NK cells compared with CXCR2- NK cells, but it did not differ among NGFR-transduced cells. CXCL8-induced CXCR2 signaling, however, did not change CD11a expression or activate a high-affinity conformation of LFA-1 on CXCR2-transduced NK cells (data not shown). As a result of the increased expression of adhesion molecules, CXCR2+ NK cells formed 53 ± 21% more conjugates with K562 cells than CXCR2- NK cells (*p* = 0.0128) (Fig. 3b-c). However, these results were not observed for NGFR-transduced NK cells. Blocking CD11a with antibodies reduced conjugate formation to background levels (Additional file 3: Figure S3). Although CXCR2+ NK cells formed conjugates at a higher rate compared with CXCR2- NK cells, no difference in their degranulation against K562 cells was observed (Fig. 3d).

### CXCR2-transduced NK cells show increased calcium mobilization in the presence of CXCR2 ligands

**Fig. 4:**
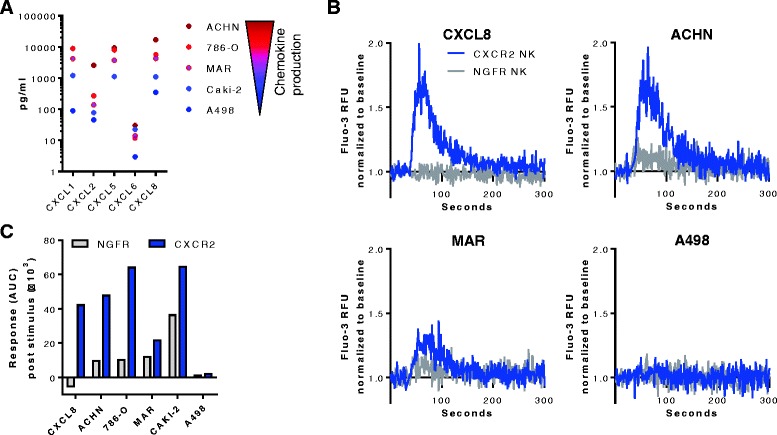
Calcium mobilization in transduced NK cells. **a** Multiplex analysis of CXCR2 ligands in the supernatants of the RCC cell lines ACHN, 786-O, MAR, CAKI-2, and A498. CXCL1 in ACHN cells was above the quantification limit of 13,990 pg/mL, CXCL5 in A498 cells was below the quantification limit of 603 pg/mL. **b** Calcium mobilization in CXCR2- and NGFR-transduced NK cells stimulated with recombinant CXCL8 (50 ng/mL) or supernatant from the RCC cell lines ACHN, MAR or A498. Values are Fluo-3 relative fluorescent units (RFU) normalized to the baseline prior to the addition of stimuli. **c** Calcium response in CXCR2- and NGFR-transduced NK cells after addition of the stimuli calculated as the normalized area under curve (AUC)

To determine if the ectopic CXCR2 receptor was functional and would respond to cognate ligands produced by RCC cell lines, the mobilization of calcium from the endoplasmatic reticulum into the cytosol was analyzed. Multiplex analysis revealed that ACHN cells produced the highest levels of the analyzed CXCR2 ligands, while A498 cells produced overall the least amount of CXCR2 ligands (Fig. 4a). CXCL1, CXCL5 and CXCL8 were overall the most abundant chemokines present in the RCC tumor supernatants. For the calcium flux assay, NK cells transduced with CXCR2 or the control NGFR were stimulated with recombinant CXCL8 or conditioned RCC tumor supernatants. CXCR2-transduced, but not control NK cells responded to CXCL8 with a rapid increase in intracellular calcium that dissolved over time (Fig. 4b). Supernatants from cell lines with high chemokine production (ACHN, 786-O) induced a stronger calcium flux in CXCR2-transduced NK cells than supernatants with lower chemokine levels (Caki-2, MAR, A498) (Fig. 4b-c and Additional file 4: Figure S4). Importantly, NGFR-transduced NK cells did not respond to stimulation with CXCL8 or the RCC supernatants. NK cells transduced with both CXCR2 and NGFR showed a similar increase in calcium when stimulated with ionomycin, indicating that their maximum capacity to release calcium was comparable (Additional file 4: Figure S4).

### CXCR2-transduced NK cells have an increased ability to migrate along a CXCR2 ligand gradient

**Fig. 5:**
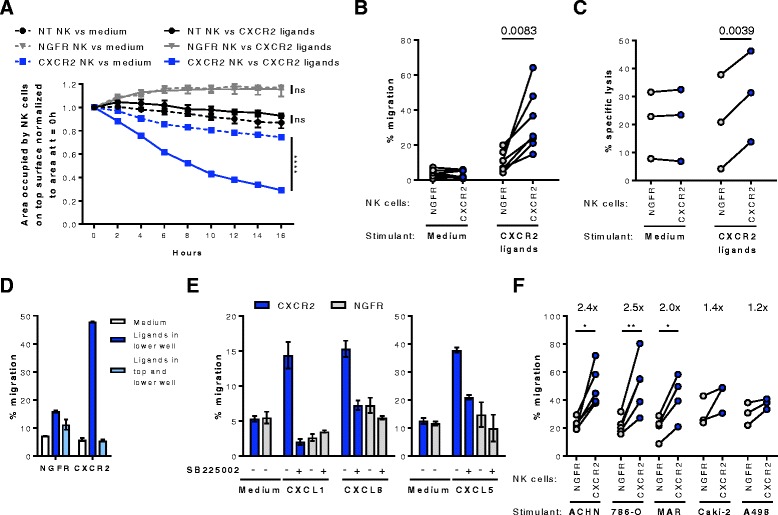
CXCR2-specific migration of CXCR2-transduced NK cells. **a** Time course of the migration of non-transduced (NT), NGFR- or CXCR2-transduced NK cells toward a pool of recombinant CXCL1, CXCL2, CXCL3, and CXCL8. Cell migration was analyzed with the IncuCyte ZOOM live cell imager and expressed as area occupied by NK cells on the top surface normalized to the initial top value (*n* = 4 per condition). Repeated measures two-way ANOVA was applied to analyze data. **b** Transwell migration assay of NT, NGFR- or CXCR2-transduced NK cells toward a pool of the recombinant CXCR2 ligands CXCL1, CXCL2, CXCL3, CXCL5, CXCL6, CXCL7, and CXCL8. The number of migrated cells was determined by automatic counting using a flow cytometer from three technical replicates per experiment (*n* = 7). **c** NK cell-mediated cytotoxicity against K562 cells after transwell migration toward medium with or without recombinant CXCR2 ligands (*n* = 3). **d** Equal concentrations of CXCR2 ligands were added to the upper and lower chambers of a transwell assay of NGFR- and CXCR2-transduced NK cells. Results are representative of three experiments with two technical replicates. **e** Transwell assay of NGFR- and CXCR2-transduced NK cells toward recombinant CXCL1, CXCL8 and CXCL5 following pre-incubation with the selective CXCR2 inhibitor SB225002. Results are representative of three experiments for CXCL1 and CXCL8 and of two experiments for CXCL5 with two technical replicates. **f** Transwell assay of NGFR- and CXCR2-transduced NK cells toward supernatants from RCC cell lines. Data are mean values from three technical replicates per experiment (*n* = 4 for ACHN, 786-O, and MAR; *n* = 3 for Caki-2 and A498). Percent migrated cells are calculated based on total cell input. *, *P* < 0.05, **, *P* < 0.01 and ****, *P* < 0.0001

To investigate if CXCR2-transduced NK cells had an increased ability to migrate toward RCC tumors that produce CXCR2 ligands, two independent chemotaxis assays were performed. A microscopy based assay that allows measurement of cell migration in real-time demonstrated that within 16 h, 71% of CXCR2-transduced NK cells migrated from the area on the top of a porous membrane to the bottom chamber that contained pooled CXCR2 ligands, whereas less than 10% of non-transduced or control NGFR-transduced NK cells migrated (Fig 5a). There was 45% more migration of CXCR2-transduced NK cells toward CXCR2 ligands was than to medium (*p* < 0.0001), while there was no significant ligand-induced migration for non-transduced or NGFR-transduced NK cells. These findings were corroborated in transwell assays showing that 3.4 times more NK cells transduced with CXCR2 than those transduced with NGFR migrated toward recombinant CXCR2 ligands (*p* = 0.0083) (Fig. 5b). The enhanced migration along the CXCR2 gradient resulted in a significantly higher lysis of K562 target cells by CXCR2-transduced NK cells in contrast to NGFR-transduced NK cells (Fig. 5c). When CXCR2 ligands were present in both the upper and lower chambers, the migration of CXCR2-transduced NK cells was abrogated, confirming the enhanced migration to be ligand-specific chemotaxis rather than increased chemokinesis (Fig. 5d). To confirm that the migration was specifically dependent on CXCR2, NK cells were incubated with the selective CXCR2 inhibitor SB225502. Indeed, in the presence of SB225502, the migration of CXCR2-transduced NK cells toward CXCL1, CXCL8 and CXCL5 was reduced to background levels, similar to those observed for control NGFR-transduced cells (Fig. 5e). Furthermore, conditioned medium from the RCC cell lines ACHN, 786-O and MAR that secrete high levels of CXCR2 ligands enhanced the migration of CXCR2-transduced NK cells 2- to 2.5-fold compared with NGFR-transduced NK cells (Fig. 5f). By contrast, low-chemokine containing supernatant from Caki-2 and A498 cells did not induce a significant increase in migration by CXCR2-transduced NK cells. For all assessed RCC tumor supernatants, the CXCL1 but not CXCL8 concentration correlated significantly (*p* = 0.014) with the migration of CXCR2-transduced NK cells relative to NGFR-transduced NK cells (Additional file 5: Figure S5).

## Discussion

In this study, we show that genetic modification of human NK cells to re-express the chemokine receptor CXCR2 conferred the ability to specifically migrate to RCC tumor-derived CXCR2 ligands resulting in increased killing of target cells.

CXCR2 ligands promote several processes important for tumorigenesis, including angiogenesis, survival and migration. We detected a concentration gradient for the CXCR2 ligands CXCL1, CXCL2, CXCL5, CXCL6 and CXCL8 between the plasma and the tumor tissues of patients with primary RCC. These gradients are the prerequisites for lymphocytes expressing the CXCR2 chemokine receptor to traffic to the tumor site. With the exception of CXCL5 that was largely not detectable in the plasma of primary RCC patients, our findings confirm previous reports describing the presence of CXCL1, CXCL3, CXCL5 and CXCL8 in the plasma and tumors of patients with metastatic RCC [21]. Importantly, CXCL5 levels correlated with the intratumoral infiltration of CXCR2-positive NK cells in our patient cohort. CXCL5 is mainly an epithelial-cell derived chemokine found in many tumors, and while several immune cells types, including neutrophils and macrophages, produce CXCL5 in the context of inflammation [22], there have been to our knowledge no reports showing CXCL5 secretion by tumor-infiltrating immune cells. López-Lago et al. demonstrated in mice that CXCL5 and CXCL8 secreted by RCC tumors constrain their ability to form pulmonary metastases through the recruitment and anti-tumor activity of neutrophils [24]. Moreover, low CXCL5 expression was associated with more aggressive tumor growth and ability to metastasize in a rat model of colorectal cancer and a poor prognosis in patients [25]. These and our findings suggest that the role of CXCL5 needs to be interpreted depending on the tumor context as this chemokine can lead to the infiltration of leukocytes that are able to mount an anti-tumor response.

Because efficient homing of lymphocytes to tumor sites is an important prerequisite for effective anti-tumor immune responses, expression of the CXCR2 chemokine receptor on adoptively transferred NK cells would be advantageous to exploit the described chemokine composition in the tumor microenvironment. CXCR2 expression on NK cells is not well established; while Campbell et al. observed low levels of CXCR2 on a subset of peripheral blood NK cells [26], Inngjerdingen et al. did neither detect CXCR2 expression on fresh nor on in vitro activated NK cells [27]. We found, however, that while circulating NK cells from healthy donors expressed CXCR2, they rapidly lost CXCR2 expression upon in vitro culture and expansion. Moreover, tumor-infiltrating NK cells from RCC patients had reduced CXCR2 expression compared with NK cell in the peripheral blood. It has been shown that upon short-term exposure to CXCL8, CXCR2 is internalized to be recycled, while upon prolonged exposure, it is degraded [28]. This could explain the down-modulation of CXCR2 in the ligand-rich tumor microenvironment, a phenomenon we have observed by exposing CXCR2-positive NK cells to recombinant CXCL8 (data not shown). While CXCR2 ligands were not added to the ex vivo expansion cultures of healthy NK cells, we cannot exclude the presence of CXCR2 ligands or other molecules in the human serum that could have led to a specific or unspecific down-regulation of CXCR2 on NK cells. Nonligand stimuli have been shown to irreversibly down-regulate CXCR2 surface levels on neutrophils though the induction of ADAM17 metalloproteinase activity [29]. While a specific ADAM17 inhibitor did not prevent the reduction of CXCR2 on NK cells (data not shown), it could have been mediated by other matrix metalloproteinases.

To overcome this issue, we genetically engineered human expanded NK cells to re-express CXCR2 in order to enhance their migration toward CXCR2-ligand expressing tumors such as RCC. Similar to murine and human T cells [30, 31], we did not observe any changes in NK cell effector functions upon retroviral transduction with chemokine receptors.

Despite no changes in NK cell effector functions, CXCR2-transduced NK cells had a significantly higher expression of CD2, a costimulatory receptor in NK cells and T cells [32], compared with non-transduced NK cells. As CD2 was recently reported to synergize with CD16 and stimulate antibody-dependent cellular cytotoxicity in NK cells [33], increased CD2 expression can be seen as an additional advantage of using CXCR2-transduced NK cells for adoptive cell therapy.

LFA-1 is a key mediator of the firm adhesion and the formation of the immunological synapse by cytotoxic lymphocytes [34]. We found that NK cells that had incorporated the CXCR2 or NGFR transgene (CXCR2+ or NGFR+) had a significantly higher expression of CD11a, the α-subunit of LFA-1, compared with NK cells that had not inserted the transgene (CXCR2- or NGFR-). The likely explanation for this observation is that NK cells with a high LFA-1 expression were more efficiently infected by the retrovirus as has been shown in T cells infected with the human immunodeficiency virus type 1 bearing host-derived ICAM-1 [35]. Consequently, CXCR2+ NK cells formed more CD11a-dependent conjugates with K562 cells than CXCR2- NK cells, a difference not observed for NGFR-transduced NK cells. However, there were no differences in degranulation against K562 cells between CXCR2+ and CXCR2- NK cells. This is in agreement with Bryceson et al. who have shown that in resting NK cells, cytotoxic granule polarization is induced by LFA-1 signaling, whereas degranulation is an LFA-1 independent event [36]. By contrast, IL-2 activated NK cells were able to lyse ICAM-1 coated insect target cells though LFA-1 signaling alone [37]. The latter would suggest that expanded CXCR2+ NK cells are more potent at killing target cells. However, tumor cells, such as K562, express a variety of ligands engaging a wide range of receptors that can activate the redundant pathways for NK cell degranulation and cytotoxicity.

We confirmed that the signaling machinery of the ectopic CXCR2 receptor was functional as it could induce calcium mobilization, one of the first steps in G-protein coupled receptor signaling, in CXCR2-transduced NK cells upon stimulation with recombinant CXCL8 as well as with RCC tumor-derived supernatants containing CXCR2 ligands. While NGFR-transduced cells did not respond to CXCL8, they released calcium upon stimulation with tumor supernatants, albeit 2 to 6 times less than CXCR2-transduced NK cells. This release was likely due to other chemokines secreted by RCC cells acting on NK cell chemokine receptors. For example, the tumor supernatants contained low levels of CXCL9, CXCL10 and CXCL11 (data not shown) that bind to CXCR3, a chemokine receptor known to be upregulated on NK cells upon activation and expansion [20].

The transduction with CXCR2 conferred to the NK cells a significantly (1.8- to 5.9-fold) increased migration ability to recombinant CXCR2 ligands compared with NGFR transduction. The enhanced trafficking along the CXCR2 gradient resulted in a significantly higher lysis of K562 target cells by NK cells transduced with CXCR2 compared with those transduced with NGFR. In addition, RCC supernatant from cell lines producing high amounts of CXCR2 ligands induced 2- to 2.5-fold increased migration of CXCR2-transduced NK cells compared with NGFR-transduced NK cells. This increase in migration was dependent on the concentration of CXCL1, but not CXCL8 in the tumor supernatants. While CXCL1 and CXCL8 have a similar potency of 5 nM and 4 nM, respectively [38], CXCL1 was more abundant in 80% of the assayed supernatants, which could explain its greater impact on NK cell migration.

Other studies have recently addressed the issue of lymphocyte homing as a key requirement for an effective anti-tumor immune response by modifying either the chemokine composition at the tumor site or the chemokine receptor repertoire of adoptively transferred lymphocytes. While increased infiltration of T and NK cells has been observed in tumors with a local production of CCL5, CXCL10 or CX3CL1, these chemokines had to be artificially introduced into the tumor microenvironment by intratumoral injections of chemokine-encoding DNA plasmids and adenoviral vectors or chemokine-stimulating cytokines such as IFN-γ [20, 39, 40]. Taking for the first time advantage of the chemokines present at the targeted site, Kershaw et al. transduced activated human T cells with the chemokine receptor CXCR2 and observed improved migration toward melanoma supernatants in vitro [41]. Subsequent studies have shown increased infiltration and anti-tumor responses of adoptively transferred mouse and human T cells engineered to express, for instance, CCR4, CXCR2 and CCR2 in transplantable mouse models of Hodgkin’s lymphoma, melanoma and mesothelioma, respectively [30, 31, 42]. With respect to NK cells, transient expression of CCR7 in human NK cells acquired by either mRNA electroporation or trogocytosis has been shown to augment their migration to CCL19 in vitro and to lymph nodes in athymic nude mice, respectively [43, 44]. Moreover, transduction of the YTS NK cell line (containing an EGFR-specific chimeric antigen receptor) with CXCR4 has been shown to enhance infiltration in glioblastoma xenograft models overexpressing CXCL12, resulting in improved survival [45]. Our study further advances the understanding how NK cell infiltration of tumors can be enhanced and shows, to our knowledge for the first time, stable engineering of human primary NK cells to express a chemokine receptor, thereby improving their migration. However, further research using in vivo models is needed to corroborate our findings and address how infused NK cells overcome challenges such as migration through the blood stream, extravasation into the tumor tissue and interplay with the tumor microenvironment to achieve an anti-tumor effect.

## Conclusions

In order to increase the success of NK cell-based therapies of solid tumors, it is of great importance not only to maintain optimal in vivo proliferation and cytotoxic activity, but also to promote homing of NK cells to the tumor site. In this study, we report that increasing chemokine receptor expression on NK cells is a promising approach to augment the efficacy of adoptive cellular immunotherapies.

## Additional files

Additional file 1: Figure S1. Time course of CXCR2 expression on healthy donor NK cells in an expansion setup with EBV-LCL feeder cells and IL-2, as assessed by flow cytometry. (PDF 16 kb)

Additional file 2: Figure S2. Gating strategy and representative flow cytometric assessment of NK cell degranulation and IFN-γ production after co-culture with K562 cells. (PDF 140 kb)

Additional file 3: Figure S3. Left: Flow cytometry analysis of CD2 expression on non-transduced (NT), NGFR-and CXCR2-transduced NK cells. Middle: Flow cytometry analysis of CD2 expression of CXCR2-transduced NK cells incorporating (CXCR2+) and not incorporating the transgene (CXCR2-). Right: NGFR- and CXCR2-transduced NK cells in conjugates with K562 cells co-cultured for 0 min and 10 min with and without CD11a-blocking antibodies (*n* = 3). (PDF 73 kb)

Additional file 4: Figure S4. Calcium mobilization in CXCR2- and NGFR-transduced NK cells stimulated with supernatant from the RCC cell lines 786-O or Caki-2 or with ionomycin (200 ng/mL) as a positive control. Values are Fluo-3 relative fluorescent units (RFU) normalized to the baseline prior to the addition of stimuli. (PDF 44 kb)

Additional file 5: Figure S5. Pearson correlation of CXCL1 and CXCL8 levels in RCC tumor supernatants used in transwell assays and corresponding migration of CXCR2-transduced NK cells relative to NGFR-transduced NK cells (*n* = 20). (PDF 19 kb)

## Abbreviations

AUC: Area under curve
BMQC: 2,3,6,7-tetrahydro-9-bromomethyl-1H,5H–quinolizino(9,1-gh)coumarin
CCL: Chemokine (C-C motif) ligand
CCR: Chemokine (C-C motif) receptor
CFSE: Carboxyfluorescein succinimidyl ester
Cr: Chromium
CX3CL: Chemokine (C-X3-C motif) ligand
CX3CR: Chemokine (C-X3-C motif) receptor
CXCL: Chemokine (C-X-C motif) ligand
CXCR: Chemokine (C-X-C motif) receptor
DNAM-1: DNAX accessory molecule-1
E:T ratio: Effector-to-target ratio
EBV: Epstein-Barr virus
FBS: Fetal bovine serum
IFN: Inteferon
IL: Interleukin
LCL: Lymphoblastoid cell line
LFA-1: Leukocyte function-associated antigen 1
Mac-1: Macrophage-1 antigen
MFI: Mean fluorescence intensity
NGFR: Nerve growth factor receptor
NK: Natural killer
NT: Non-transduced
PBMC: Peripheral blood mononuclear cell
PBS: Phosphate buffered saline
pMSGV: Murine stem cell virus-based splice-gag vector plasmid
RCC: Renal cell carcinoma
RFU: Relative fluorescence unit
TRAIL: Tumor necrosis factor-related apoptosis-inducing ligand
