# Correction to: Genetic engineering of human NK cells to express CXCR2 improves migration to renal cell carcinoma

**DOI:** 10.1186/s40425-017-0292-8

**Published:** 2017-11-06

**Authors:** Veronika Kremer, Maarten A. Ligtenberg, Rosa Zendehdel, Christina Seitz, Annet Duivenvoorden, Erik Wennerberg, Eugenia Colón, Ann-Helén Scherman-Plogell, Andreas Lundqvist

**Affiliations:** 1 Department of Oncology-Pathology, Karolinska Institutet, Stockholm, Sweden; 2 Department of Molecular Oncology, The Netherlands Cancer Institute, Amsterdam, Netherlands; 3 Department of Medicine Solna, Karolinska Institutet, Stockholm, Sweden; 4 Department of Radiation Oncology, Weill Cornell Medicine, New York, NY USA; 5 Department of Oncology-Pathology, Stockholm South General Hospital, Stockholm, Sweden; 6 Department of Woman and Child Health, Karolinska Institutet, Stockholm, Sweden; 7 Department of Urology, Stockholm South General Hospital, Stockholm, Sweden; 8 Cell Therapy Institute, Nova Southeastern University, Fort Lauderdale, FL USA

Unfortunately, after publication of this article [1], it was noticed that the name of Maarten A. Ligtenberg was displayed incorrectly as Maarten Ligtenberg. The correct author list can be seen above and the original article has been updated to reflect this.
