# Supplementary figures and images for: Genetic engineering of human NK cells to express CXCR2 improves migration to renal cell carcinoma
Source: J Immunother Cancer. 2017 Sep 19;5:73. doi: 10.1186/s40425-017-0275-9 (PMC5604543; doi:10.1186/s40425-017-0275-9)

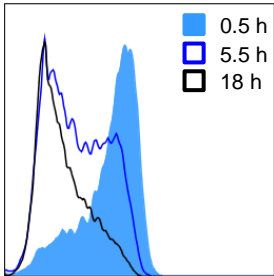

CXCR2

Supplement: Supplementary file 1 — Time course of CXCR2 expression on healthy donor NK cells in an expansion setup with EBV-LCL feeder cells and IL-2, as assessed by flow cytometry. (PDF 16 kb) [file 40425_2017_275_MOESM1_ESM.pdf]

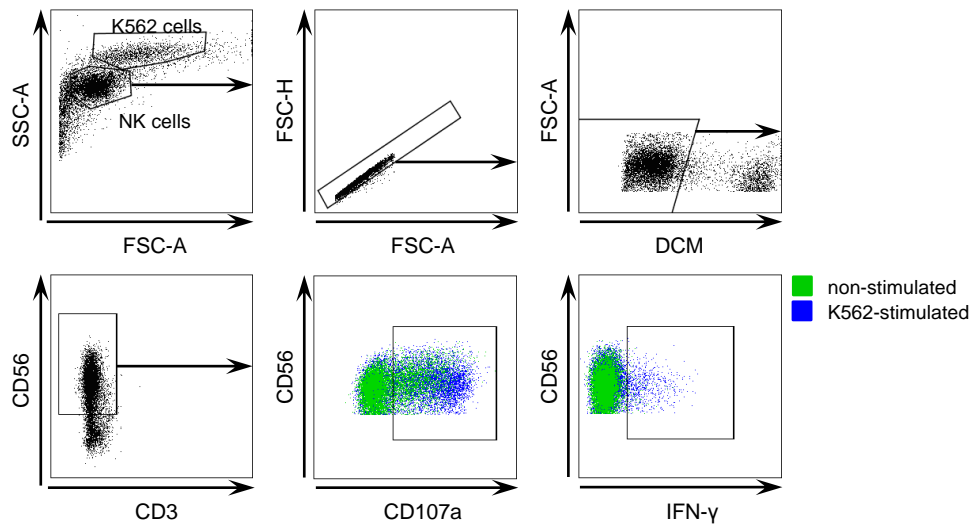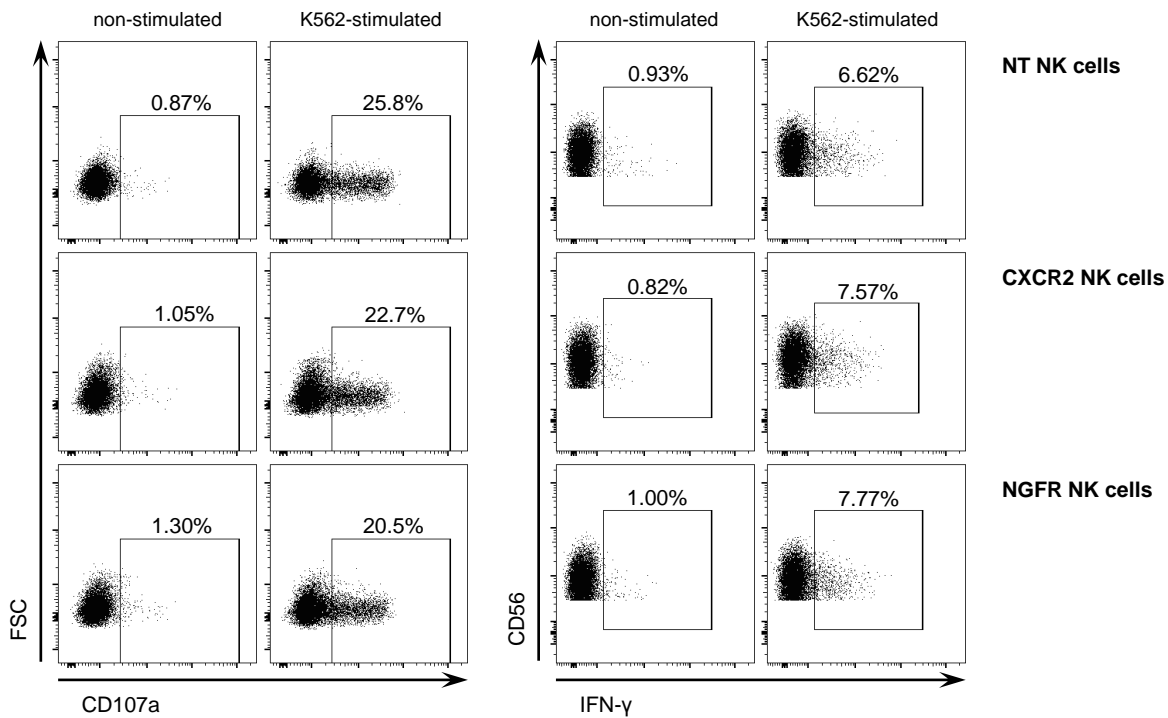

Supplement: Supplementary file 2 — Gating strategy and representative flow cytometric assessment of NK cell degranulation and IFN-γ production after co-culture with K562 cells. (PDF 140 kb) [file 40425_2017_275_MOESM2_ESM.pdf]

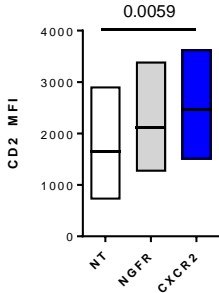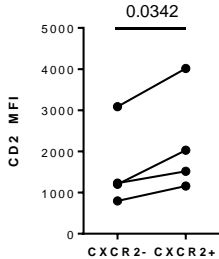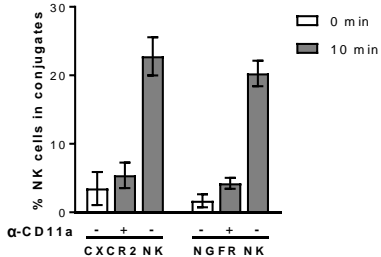

Supplement: Supplementary file 3 — Left: Flow cytometry analysis of CD2 expression on non-transduced (NT), NGFR-and CXCR2-transduced NK cells. Middle: Flow cytometry analysis of CD2 expression of CXCR2-transduced NK cells incorporating (CXCR2+) and not incorporating the transgene (CXCR2-). Right: NGFR- and CXCR2-transduced NK cells in conjugates with K562 cells co-cultured for 0 min and 10 min with and without CD11a-blocking antibodies (n = 3). (PDF 73 kb) [file 40425_2017_275_MOESM3_ESM.pdf]

**786-O**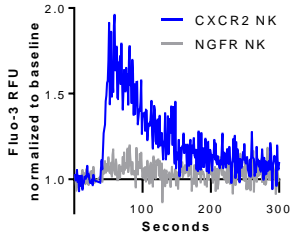**Caki-2**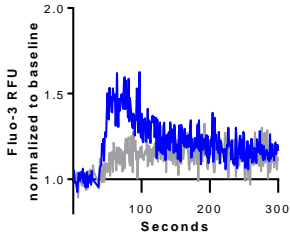**Ionomycin**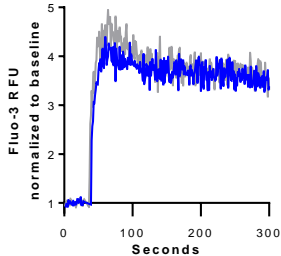

Supplement: Supplementary file 4 — Calcium mobilization in CXCR2- and NGFR-transduced NK cells stimulated with supernatant from the RCC cell lines 786-O or Caki-2 or with ionomycin (200 ng/mL) as a positive control. Values are Fluo-3 relative fluorescent units (RFU) normalized to the baseline prior to the addition of stimuli. (PDF 44 kb) [file 40425_2017_275_MOESM4_ESM.pdf]

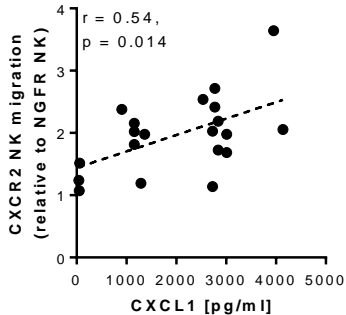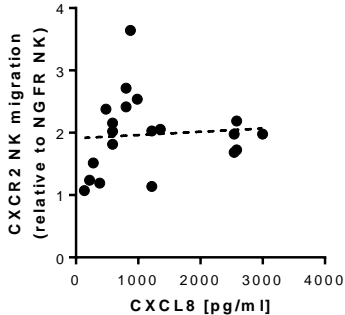

Supplement: Supplementary file 5 — Pearson correlation of CXCL1 and CXCL8 levels in RCC tumor supernatants used in transwell assays and corresponding migration of CXCR2-transduced NK cells relative to NGFR-transduced NK cells (n = 20). (PDF 19 kb) [file 40425_2017_275_MOESM5_ESM.pdf]
